# The role of peptide YY in gastrointestinal diseases and disorders

**DOI:** 10.3892/ijmm.2012.1222

**Published:** 2012-12-21

**Authors:** MAGDY EL-SALHY, TAREK MAZZAWI, DORIS GUNDERSEN, JAN GUNNAR HATLEBAKK, TRYGVE HAUSKEN

**Affiliations:** 1 Section for Gastroenterology, Department of Medicine, Stord Helse-Fonna Hospital, Stord;; 2 Department of Research, Helse-Fonna, Haugesund;; 3 Section for Gastroenterology, Institute of Medicine, University of Bergen, Bergen, Norway

**Keywords:** diabetes gastroenteropathy, chronic idiopathic slow transit constipation, familial amyloidosis with polyneuropathy, irritable bowel syndrome, inflammatory bowel disease, lymphocytic colitis, peptide YY, serotonin

## Abstract

Peptide YY (PYY) is affected in several gastrointestinal diseases and disorders. Changes in PYY appear to be an adaptive response to alterations in pathophysiological conditions caused by the disease. This applies to gastrointestinal diseases/disorders such as irritable bowel syndrome, inflammatory bowel disease, celiac disease, systemic sclerosis, and post-intestinal resection. By contrast, the changes in PYY in chronic idiopathic slow transit constipation (CST) seem to be of a primary nature, and may be one etiological factor of the disease. Abnormalities in PYY seem to contribute to the development of symptoms present in irritable bowel syndrome, inflammatory bowel disease, gastroenteropathy in long-standing diabetes and CST. The changes in PYY could, however, be favorable in some gastrointestinal disorders such as celiac disease, systemic sclerosis and post-intestinal resection state. Investigating changes in PYY in gastrointestinal diseases/disorders could be beneficial in clinical practice, where a receptor agonist or an antagonist can be used as a drug, depending on the condition. Similar to other neuroendocrine peptides/amines of the gut, PYY has broad physiological/pharmacological effects: it can bind to and activate several receptors with independent actions. Thus, in order to use PYY as a drug, receptor-specific agonists or antagonists need to be developed.

## Contents

1. Introduction
2. Irritable bowel syndrome
3. Inflammatory bowel disease
4. Chronic idiopathic slow transit constipation
5. Celiac disease
6. Diabetes gastroenteropathy
7. Colorectal carcinoma
8. Neuromuscular and system diseases
9. Surgical resection of stomach and intestine
10. Conclusion

## 1. Introduction

Peptide YY (PYY) was originally isolated from porcine gut (1–3). PYY has close molecular similarities to neuropeptide Y (NPY) and pancreatic polypeptide (PP) (2–4), which has led to the suggestion that they be grouped in a family: the ‘PP-related peptides’ family (5). In humans, PYY has been found localized in endocrine cells in the colon (6). Further studies on humans have shown that PYY immunoreactive cells occur in the ileum, colon, and rectum, with the highest density in the rectum (7). Furthermore, PYY immunoreactivity has been localized ultrastructurally in large intestinal H(L)-cells, whose secretory product was previously unknown (7). PYY endocrine cells occur in the gastrointestinal mucosa of representatives of all the vertebrate classes, i.e. cartilaginous and bony fish, amphibians, reptiles, birds and mammals (8–15). The topo-graphic distribution of PYY in the gastrointestinal tract differs, however, in different animals. Thus, in primates, PYY cells occur in the ileum and large intestine with the highest concentration in the rectum, whereas in rats, PYY cells occur in all parts of the small and large intestine, and in fish, reptiles, and amphibians, in the stomach and upper part of the small intestine. Ontological studies have shown PYY cells appear early in the gastrointestinal tract of the embryo (10,12,16). Although PYY cell density is not affected by aging (16), it is abnormal in several gastrointestinal diseases and disorders (17).

The release of PYY from intestinal endocrine cells is stimulated by intraluminal nutrients, lipids, short-chain fatty acids, glucose, amino-acids, and bile salts. PYY release can also be mediated via a neural reflex involving the vagus nerve, as well as by other gut neuroendocrine peptides such as vasoactive intestinal peptide (VIP), cholecystokinin (CCK), gastrin, and glucagon-like peptide-1 (GLP-1) (18).

PYY is one of the major anorexigenic gastrointestinal neuroendocrine peptides (19). Upon release, PYY is metabolized by dipeptidyl peptidase-IV (DPP-IV) to PYY (3–36), which crosses the blood-brain barrier. There, it binds to Y2 receptors on NPY neurons in the arcuate nucleus of the hypothalamus. Thus, it eliminates the tonic inhibition on proopiomelanocortin (POMC) neurons with subsequent satiation (20–22). PYY plays an important role in regulating gastrointestinal motility and absorption of water and electrolytes (23–25). These functions regulated by PYY are disturbed in several gastrointestinal diseases and disorders (17). It is not surprising, therefore, that abnormalities in PYY have been reported in gastrointestinal diseases and disorders. The aim of the present review was to provide an overview of the changes in PYY in some gastrointestinal diseases and disorders, and their possible clinical implications.

## 2. Irritable bowel syndrome

Irritable bowel syndrome (IBS) is a chronic common syndrome affecting 5–20% of the world’s population. IBS symptoms include diarrhea, constipation, or a combination of the two, and abdominal pain or discomfort as well as abdominal distension. IBS is not known to be associated with the development of serious disease or with excess mortality. IBS, however, reduces considerably the quality of life. IBS patients are a substantial concern in both primary and secondary care, and the annual cost in the USA, both direct and indirect, for the management of patients with IBS is estimated at 15–30 billion USD (26). The pathogenesis of IBS is not completely known, but it appears to be multifactorial. Evidence shows that the following factors play a central role in the pathogenesis of IBS: heritability and genetics, environment and social learning, dietary or intestinal microbiota, low-grade inflammation, and disturbances in the neuroendocrine system (NES) of the gut (26). A subset of IBS patients, with no previous gastrointestinal complaints, have a sudden onset of IBS symptoms following gastroenteritis. This subset is called post-infectious IBS (PI-IBS). PI-IBS, however, has also been reported following non-gastrointestinal infections such as respiratory, urinary tract and skin infections (26).

**Figure 1:**
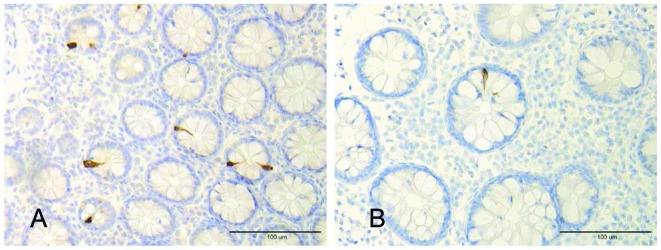
PYY-immunoreactive cells in the colon of (A) a healthy volunteer and (B) a patient with IBS.

In the large intestine of sporadic IBS patients, PYY cell density was found to be low in both IBS-constipation and IBS-diarrhea patients (Fig. 1) (27). In the large intestine of the same patients, serotonin cell density was reduced and the mucosal 5-HT concentration was also reported to be low (28). In the duodenum of IBS patients, the number of CCK cells was also low (29). Serotonin acts on 5-HT1p receptors, which are located on a subset of inhibitory motor neurons of the myenteric plexus and relax the stomach via a nitrergic pathway, delaying gastric emptying (26). The primary targets of serotonin are the mucosal projections of primary afferent neurons, which transmit the sensation of nausea and discomfort to the central nervous system, and the mucosal projections of intrinsic primary afferent neurons, which initiate peristaltic and secretory reflexes (30–35). Serotonin also stimulates the secretion of chloride and water from the small intestine by acting through 5-HT3 and 5-HT4 receptors (36–40). The low density of serotonin cells is likely to reduce motility and secretion of chloride and water in the colons of patients with IBS. As compensation for this, PYY is reduced. As previously mentioned, CCK stimulates the release of PYY. Moreover, low CCK would result in low bile salts, which are stimulatory for PYY secretion. Thus, a low CCK could contribute to the low density of large intestinal PYY cells in IBS patients. The findings of genetic in transmission pathways of serotonin and CCK (41–44) support the assumption that the change in colonic PYY cells is secondary to changes in serotonin and CCK.

In contrast to sporadic IBS, PYY cell numbers are reported to be increased in the large intestine of PI-IBS patients (45). Serotonin and CCK cell densities are also increased in these patients (45–50). As low-grade inflammation has been reported in PI-IBS, the increase in cell density of CCK and serotonin cells appears to be a result of an interaction with immune cells, as described below.

Therefore, PYY is affected in IBS and may play a role in its symptomology. These changes, however, seem to be secondary to the changes in CCK and serotonin.

## 3. Inflammatory bowel disease

Inflammatory bowel disease (IBD) comprises two distinct disorders with independent clinicopathologies of unknown etiology. These diseases, ulcerative colitis (UC) and Crohn’s disease (CD), are fairly distinct in their organ specificity and their histopathological characteristics. The onset of IBD occurs most often at the age of 20–30 years. Thus, IBD represents an important public health problem as it affects young individuals, interfering with the patient’s education, working abilities, social life, and quality of life. These diseases are chronic conditions whose clinical courses vary considerably, with frequent relapses or chronic active disease in some patients, whereas some have years of virtually complete remission. In addition to UC and CD, another inflammatory bowel disease is included in this category, microscopic colitis (MC). MC is also a chronic condition, characterized by watery diarrhea with normal radiologic and endoscopic findings. However, histopathological examinations of the colon reveal abnormal histology (51). This abnormality is of two distinctive types: lymphocytic colitis (LC) and collagenous colitis (CC) (51).

**Figure 2:**
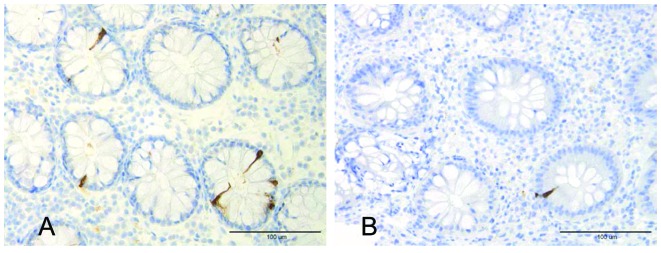
Photomicrograph of PYY-immunoreactive cells in the colon of (A) a healthy volunteer and (B) a patient with ulcerative colitis.

**Figure 3:**
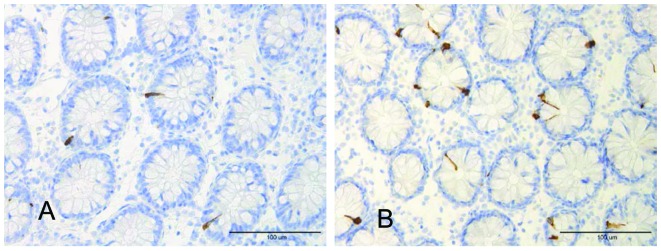
PYY-immunoreactive cells in the colon of (A) a healthy volunteer and (B) a patient with lymphocytic colitis.

Colonic PYY cell area was found to be decreased in patients with UC and CD (Fig. 2), whereas those with serotonin and enteroglucagon immunoreactivities were elevated (52). As enteroglucagon and PYY are colocalized in the same colorectal endocrine cell type (L-cells) (53–55), it appears that this cell increased its expression of enteroglucagon, and reduced expression of PYY. In the ileal mucosa of patients with CD, PYY cell density was decreased, as was that of serotonin (56). In one study, serotonin cell density in rectal biopsies from patients with UC was found to be elevated (57), whereas another study showed it to be decreased (28). In rectal biopsies from patients with UC, mucosal serotonin, tryptophan hydroxylase 1 messenger RNA, serotonin transporter messenger, and serotonin transporter were all reduced (28). It has also been shown that patients with LC have high densities of colonic PYY and serotonin cells (58) (Fig. 3).

In an experimental animal model of colitis (IL-2 knockout mice), PYY and serotonin cell densities were decreased in mice with colitis, whereas enteroglucagon remained unchanged (59). In another animal model (rats treated with dextran sulfate sodium), the PYY density decreased in both the small and large intestines (60).

Increasing evidence shows that inflammation and immune cells interact with the NES of the gut, which controls and regulates gastrointestinal motility and sensitivity (61). Thus, serotonin secretion by enterochromaffin (EC) cells can be enhanced or attenuated by secretory products of immune cells such as CD4^+^ T lymphocytes (62). Furthermore, serotonin modulates the immune response (62). The EC cells are in contact with or very close to CD3^+^ and CD20+ lymphocytes, and several serotonergic receptors have been characterized in lymphocytes, monocytes, macrophages, and dendritic cells (63). Moreover, immune cells in the small and large intestines show receptors for substance P and VIP (59).

Based on the above-mentioned interaction between immune and serotonin cells, it may be assumed that the changes in serotonin cells are caused by the inflammatory process. It further seems that the changes in PYY cells in IBD are secondary to changes in serotonin cells.

## 4. Chronic idiopathic slow transit constipation

Chronic idiopathic slow transit constipation (CST) is a common clinical problem. This condition is characterized by chronic severe constipation, which is not alleviated by bulking agents, prokinetic drugs or other laxative treatments. These patients require enema for defecation (64,65). Histopathological examination fails to identify any abnormality in the colon of these patients (64,65). However, slow colonic transit and motility disorders of the colon and rectum have been found in these patients (66–70).

PYY cells have been reported to be increased compared to controls in the ascending colon of patients with CST (71). In another study from our laboratory, however, the number of colonic PYY cells was unaffected (72). The concentration of PYY in colonic tissue extracts from patients with CST has been reported to be high (73), but basal and peak plasma PYY levels have been reported to be unaffected (74). These results initially appear to be contradictory. However, taking CST patients as individuals instead of as a group, and analyzing the neuroendocrine peptide profile in the colon of each individual, revealed that a disturbance in the neuroendocrine system is the most probable cause for the disease, and that this disturbance affects different neuroendocrine peptides in different patients (73). This seems to explain the contradiction found in different studies.

The increase in the number of colonic PYY cells and PYY synthesis seems to be primary and may be one of the etiologic factors for CST. Consequently, this increase would increase absorption and decrease secretion of water and electrolytes, strengthening the ileal brake and inhibiting intestinal motility, which leads to constipation.

## 5. Celiac disease

Celiac disease is associated with derangement of the architecture of the small intestinal mucosa in the form of villus atrophy, increased crypt length, and increased volume of lamina propria (75). Several changes in the small intestinal endocrine cells have been reported (75). Basal and postprandial plasma levels of PYY are elevated in patients with celiac disease (76,77). PYY levels have been found to be inversely correlated with the concentration of serum folate acid (77). These elevated levels of PYY have been reported to normalize within 8 months on a gluten-free diet (77).

The changes in the endocrine cells of the small intestine in patients with celiac disease are considered to be a selective process to meet the new demands exerted by the marked decrease in intestinal absorptive area; these changes contribute to the manifestation of the symptoms seen in patients with celiac disease, such as diarrhea and steatorrhea (75). This could be caused by incomplete digestion of ingested food and its rapid elimination from the intestine (75). The elevated levels of circulating PYY in patients with celiac disease appear to be involved in this hormonal process, and are probably a response to diarrhea and steatorrhea, an attempt to slow down the intestinal transit time and increase intestinal absorption. The finding that patients with diarrhea due to other causes, such as chronic destructive pancreatitis and infective gastroenteritis, also have high plasma levels of PYY support this assumption (78).

## 6. Diabetes gastroenteropathy

Gastrointestinal symptoms, such as nausea and vomiting, diarrhea, constipation, and abdominal pain, are common in patients with diabetes mellitus (79–85). These symptoms are considered to be caused by gastrointestinal dysmotility and secretion/absorption disturbances (81). Abnormalities in PYY have been reported in patients with diabetes type 1, and in animal models of human diabetes.

Rectal PYY cells were investigated in patients with a long duration (13–48 years) of diabetes type 1, with organ complications and gastrointestinal symptoms, such as nausea, vomiting and diarrhea; they also had slow gastric emptying. The number of rectal PYY cells in these patients was found to be significantly higher than that in healthy volunteers (86).

In animal models of human diabetes type 1, i.e. non-obese diabetic (NOD) mice, the number of colonic PYY cells was reduced in diabetic, but not in pre-diabetic NOD mice (83–86). Radioimmunoassay of tissue extracts showed, however, low concentrations of colonic PYY in both pre-diabetic and diabetic mice (87). It seems that the synthesis of PYY decreased prior to the onset of diabetes, although the number of PYY cells was unaffected. Once the diabetic state is established, even the number of PYY cells declines. This animal model exhibits slow gastric emptying, fast gastrointestinal transit, and diarrhea (88–90).

The studies of PYY in animal models of human diabetes type 1 and patients with diabetes appear to yield contradictory results. Thus, whereas the number of PYY cells and the concentration of PYY in the large intestine of NOD mice was low, the number of rectal PYY cells was high in patients with diabetes type 1. This discrepancy may be due to the NOD mouse model not being completely similar to human diabetes type 1, or to the difference in the segment of large intestine being studied (the colon in NOD mice and the rectum in diabetic patients). It is most likely, however, that the difference was caused by the difference in the duration of the diabetic state. Thus, whereas the NOD mice were investigated shortly after the onset of diabetes, patients were studied after a long duration of diabetes.

PYY has been studied in two animal models of human diabetes type 2, namely ob/ob and db/db obese diabetic mice. In ob/ob mice, the number of colonic PYY cells and the tissue concentrations of PYY were found to be lower than those of controls (83–86,90). By contrast, the number of colonic PYY cells in db/db mice were reported to be higher than in controls (91). This disagreement in the results was explained by the difference in the duration of diabetes in ob/ob mice (83–86).

As already mentioned, PYY inhibits the secretion of fluid and electrolytes, while stimulating their absorption in the intestine. It is also a potent mediator of ileal brake, which inhibits gastric emptying and delays intestinal transit. It is possible that at the onset of diabetes type 1, hyperglycemia, as well as other factors, inhibit gastric motility and, consequently, gastric emptying. As PYY inhibits gastric emptying, the number of PYY cells and their synthesis is decreased in an attempt to compensate for the slow gastric emptying. Subsequently, when fast intestinal transit and diarrhea developed in these patients, PYY cells and, possibly, PYY synthesis, increased in response to these changes. This increase would, however, worsen the gastric emptying. In diabetes type 2, at least in animal models, PYY cells and synthesis may be decreased to compensate for the slow intestinal transit and constipation.

## 7. Colorectal carcinoma

The number of PYY cells in the colons of rats with chemically induced adenocarcinoma has been reported to be high (92). The difference in concentration of PYY in colon tissue extracts from these animals was not statistically significant, although it was higher than in controls (93). In patients with colorectal carcinoma, neither the number of PYY cells nor the concentration of PYY in the colon is affected (94–96). PYY receptors have been demonstrated in colonic adenocarcinoma cell lines; however, PYY exerts no direct growth regulatory effect on colon cancer cell lines (97–99). Collectively, these findings show that it is unlikely that PYY is involved in the development and growth of colorectal carcinoma.

## 8. Neuromuscular and system diseases

PYY cells have been studied in the large intestine in two hereditary diseases that affect either the nervous system or muscles, i.e. familial amyloidotic polyneuropathy (FAP) and myotonic dystrophy (MD) (100–103). FAP is caused by amyloid deposits of mutated transthyretin in the nervous system and other organs. Several gastrointestinal symptoms, such as constipation, nausea, vomiting, and diarrhea, are invariably present in FAP patients during the course of the disease (104–106). MD is a disease caused by a genetic defect in chromosome 19. Gastrointestinal symptoms, such as abdominal pain, nausea and diarrhea, are often encountered in MD patients (103). The gastrointestinal symptoms in both diseases are believed to be caused by gastrointestinal dysmotility (100,103). Despite this gastrointestinal dysmotility, PYY cells in the large intestine of FAP patients, both in Sweden and Japan, as well as of MD patients, were unaffected (100,102,103). It seems, therefore, that PYY does not play a significant role in these categories of patients.

Plasma levels of PYY in patients with systemic sclerosis have been found to be elevated (107). Fat malabsorption has also been found to be more common among patients with increased levels of plasma PYY. This increase in circulating PYY in patients with systemic sclerosis seems to be secondary to the diarrhea, rather than a primary cause, and is probably an attempt to slow down gastrointestinal motility.

## 9. Surgical resection of stomach and intestine

Surgical resection of the stomach and intestine is frequently used as primary treatment, or when medical therapy has failed, in several gastrointestinal diseases, such as gastric carcinoma, colorectal carcinoma, inflammatory bowel disease and CST.

Gastric resection is associated with several problems, such as dumping syndrome, reflux esophagitis, and malabsorption. The serum levels of several neuroendocrine hormones have been investigated in patients with partial distal gastrectomy or total gastrectomy. The levels of circulating PYY in these patients were elevated (108). This elevation may be an adaptation to compensate for the rapid gastric transit, and an attempt to slow it.

Basal and postprandial levels of PYY increased in the intestine adjacent to the anastomatic site after a massive (75%) small bowel resection in dog (76). This increase was observed one month after the small bowel resection and remained high throughout the six-month experiment. Circulating PYY concentrations have also been investigated in patients subjected to small bowel resection, mainly due to Crohn’s disease. Thus, in patients receiving treatment with home parental nutrition following near-total enterectomy, basal and postprandial circulating PYY have been found to be high (109). Similarly, elevated fasting serum PYY levels have been reported in patients with Crohn’s disease who had previous resections of >48 cm of the ileum (109). Circulating PYY levels have also been measured (110,111). Basal and postprandial plasma levels of PYY in patients who have undergone resection of the colon increased after the construction of a pelvic reservoir (110). The combination of an infusion of oleic acid in the ileal pouch and a meal increased PYY plasma levels, slowed gastrointestinal transit, and delayed defecation (111). Massive small bowel resection in 4-week-old piglets also resulted in an increase in colonic PYY cells (112).

Following resection of a considerable part of either the small or large intestine, PYY synthesis and release increased as an adaptive response. This response is an attempt to slow the rapid gastrointestinal transit caused by the intestinal resection.

## 10. Conclusion

PYY changes in several gastrointestinal disorders. These changes seem to be adaptive responses to the pathophysiological alterations caused by the disease. However, in some gastrointestinal disorders, such as chronic idiopathic slow transit constipation (CST), the abnormality in PYY seems to be primary and may, at least in part, be one of the causes of the disease. These abnormalities in PYY seem to contribute to the development of symptoms seen in gastrointestinal diseases/disorders such as gastroenteropathy in long-standing diabetes, inflammatory bowel disease and CST. The changes in PYY could, however, be favorable in some gastrointestinal disorders such as celiac disease, systemic sclerosis, and post-intestinal resection state.

The accumulated data regarding the changes in PYY in gastrointestinal disorders could be beneficial in clinical practice. Thus, a receptor agonist or antagonist can be used as a drug depending on the condition. Infusion of PYY in dogs increases colonic absorption of water, Na and Cl ions (113), and intraluminal administration of a synthetic analog, BIM-34004, has the same effect (114). It has been suggested that PYY or its analog can be used as clinical agents in intestinal malabsorption disorders or after bowel resection (113,114). In clinical trials, nausea and fullness are the most common side-effects of PYY (115). Similar to other neuroendocrine peptides/amines of the gut, PYY has broad physiological/pharmacological effects: it can bind to and activate several receptors with independent actions. Thus, in order to use PYY as a drug, receptor-specific agonists or antagonists need to be developed.
